# Supplementary material for: New Insights on Nucleotide Sequence Variants and mRNA Levels of Candidate Genes Assessing Resistance/Susceptibility to Mastitis in Holstein and Montbéliarde Dairy Cows
Source: Vet Sci. 2023 Jan 3;10(1):35. doi: 10.3390/vetsci10010035 (PMC9861242; doi:10.3390/vetsci10010035)
Supplement: Supplementary file 1 [file vetsci-10-00035-s001.zip › vetsci-2092873-supplementary.pdf]

```
NM_001144078.1      ACTCATGGCTGCAGAGCAGTCCCTAAAGCGAGACTAGAGGCGAAGCCCGCCAGCAGCCCC      60
HH                  ACTCATGGCTGCAGAGCAGTCCCTAAAGCGAGACTAGAGGCGAAGCCCGCCAGCAGCCCC      60
HM                  ACTCATGGCTGCAGAGCAGTCCCTAAAGCGAGACTAGAGGCGAAGCCCGCCAGCAGCCCC      60
MH                  ACTCATGGCTGCAGAGCAGTCCCTAAAGCGAGACTAGAGGCGAAGCCCGCCAGCAGCCCC      60
MM                  ACTCATGGCTGCAGAGCAGTCCCTAAAGCGAGACTAGAGGCGAAGCCCGCCAGCAGCCCC      60
                    ************************************************************

NM_001144078.1      CTCCCCTGCCATCCCGGCCTGGCCCAGATCACCCAGTTCCGAATGATGGTGCCTCTGGGA      120
HH                  CTCCCCTGCCATCCCGGCCTGGCCCAGATCACCCAGTTCCGAATGATGGTGCCTCTGGGA      120
HM                  CTCCCCTGCCATCCCGGCCTGGCCCAGATCACCCAGTTTCGAATGATGGTGCCTCTGGGA      120
MH                  CTCCCCTGCCATCCCGGCCTGGCCCAGATCACCCAGTTTCGAATGATGGTGCCTCTGGGA      120
MM                  CTCCCCTGCCATCCCGGCCTGGCCCAGATCACCCAGTTTCGAATGATGGTGCCTCTGGGA      120
                    ************************************** *********************

NM_001144078.1      CATTTTGCCAAAGGAGCCAGCCTGGACGATCTCATCGACAGCTGTGTTCAATCTTTTGAT      180
HH                  CATTTTGCCAAAGGAGCCAGCCTGGACGATCTCATCGACAGCTGTGTTCAATCTTTTGAT      180
HM                  CATTTTGCCAAAGGAGCCAGCCTGGACGATCTCATCGACAGCTGTGTTCAATCTTTTGAT      180
MH                  CATTTTGCCAAAGGAGCCAGCCTGGACGATCTCATCGACAGCTGTGTTCAATCTTTTGAT      180
MM                  CATTTTGCCAAAGGAGCCAGCCTGGACGATCTCATCGACAGCTGTGTTCAATCTTTTGAT      180
                    ************************************************************

NM_001144078.1      GCAGACGGAAACCTGTGTCGAAGTAACCAACTGTTGCAAGTCATGCTGACCATGCACCGA      240
HH                  GCAGACGGAAACCTGTGTCGAAGTAACCAACTGTTGCAAGTCATGCTGACCATGCACCGA      240
HM                  GCAGACGGAAACCTGTGTCGAAGTAACCAACTGTTGCAAGTCATGCTGACCATGCACCGA      240
MH                  GCAGACGGAAACCTGTGTCGAAGTAACCAACTGTTGCAAGTCATGCTGACCATGCACCGA      240
MM                  GCAGACGGAAACCTGTGTCGAAGTAACCAACTGTTGCAAGTCATGCTGACCATGCACCGA      240
                    ************************************************************

NM_001144078.1      ATTCTCATTTCCTCTGCTGAACTGCTCCAAAAAGTTATCACTCTCTATAAGGATGCCTTG      300
HH                  ATTCTCATTTCCTCTGCTGAACTGCTCCAAAAAGTTATCACTCTCTATAAGGATGCCTTG      300
HM                  ATTCTCATTTCCTCTGCTGAACTGCTCCAAAAAGTCATCACTCTCTATAAGGATGCCTTG      300
MH                  ATTCTCATTTCCTCTGCTGAACTGCTCCAAAAAGTTATCACTCTCTATAAGGATGCCTTG      300
MM                  ATTCTCATTTCCTCTGCTGAACTGCTCCAAAAAGTTATCACTCTCTATAAGGATGCCTTG      300
                    *********************************** ************************

NM_001144078.1      GCAAAGAATTCACCAGGGCTTTGCCTGAAGATCTGCTATTTTGTAAGGTACTGGATAACA      360
HH                  GCAAAGAATTCACCAGGGCTTTGCCTGAAGATCTGCTATTTTGTAAGGTACTGGATAACA      360
HM                  GCAAAGAATTCACCAGGGCTTTGCCTGAAGATCTGCTATTTTGTAAGGTACTGGATAACA      360
MH                  GCAAAGAATTCACCAGGGCTTTGCCTGAAGATCTGCTATTTTGTAAGGTACTGGATAACA      360
MM                  GCAAAGAATTCACCAGGGCTTTGCCTGAAGATCTGCTATTTTGTAAGGTACTGGATAACA      360
                    ************************************************************

NM_001144078.1      GAATTCTGGATCATGTTTAAGATGGATACCAGCCTGGCAAGCACCATGGA      410
HH                  GAATTCTGGATCATGTTTAAGATGGATACCAGCCTGGCAAGCACCATGGA      410
HM                  GAATTCTGGATCATGTTTAAGATGGATACCAGCCTGGCAAGCACCATGGA      410
MH                  GAATTCTGGATCATGTTTAAGATGGATACCAGCCTGGCAAGCACCATGGA      410
MM                  GAATTCTGGATCATGTTTAAGATGGATACCAGCCTGGCAAGCACCATGGA      410
                    **************************************************
```

Figure S1. *RASGRP1* gene (410-bp) demonstrative DNA sequence alignment between healthy and mastitis affected Holstein and Montbéliarde dairy cows together with the reference sequence found in GenBank gb|NM_001144078.1|. HH= Healthy Holstein; HM= Healthy Montbéliarde; MH= Mastitic Holstein; and MM= Mastitic Montbéliarde.

```
NM_001076409.1      TCAGGTCAAACTCCAGAATGGCAGAAGACGACCCGTATTTGGGAGGGCGTGAACAAATGT      60
HH                  TCAGGTCAAACTCCAGAATGGCAGAAGACGACCCGTATTTGGGAGGGCGTGAACAAATGT      60
HM                  TCAGGTCAAACTCCAGAATGGCAGAAGACGACCCGTATTTGGGAGGGCGTGAACAAATGT      60
MH                  TCAGGTCAAACTCCAGAATGGCAGAAGACGACCCGTATTTGGGAGGGCGTGAACAAATGT      60
MM                  TCAGGTCAAACTCCAGAATGGCAGAAGACGACCCGTATTTGGGAGGGCGTGAACAAATGT      60
                    ************************************************************

NM_001076409.1      TTCATCTGGATCCTCTGAATCATACAATGTTTAGTCCAGAATTATTTCAACCGGAGATGC      120
HH                  TTCATCTGGATCCTCTGAATCATACAATGTTTAGTCCAGAATTATTTCAACCGGAGATGC      120
HM                  TTCATCTGGATCCTCTGAATCATACAATGTTTAGTCCAGAATTATTTCAACCGGAGATGC      120
MH                  TTCATCTGGATCCTCTGAATCATACAATGTTTAGTCCAGAATTATTTCAACCGGAGATGC      120
MM                  TTCATCTGGATCCTCTGAATCATACAATGTTTAGTCCAGAATTATTTCAACCGGAGATGC      120
                    ************************************************************

NM_001076409.1      CACTACCAACAGCAGATGGCCCATACCTTCAAATATTAGAGCAACCTAAACAGAGAGGAT      180
HH                  CACTACCAACAGCAGATGGCCCATACCTTCAAATATTAGAGCAACCTAAACAGAGAGGAT      180
HM                  CACTACCAACAGCAGATGGCCCATACCTTCAAATATTAGAGCAACCTAAACAGAGAGGAT      180
MH                  CACTACCAACAGCAGATGGCCCATACCTTCAAATATTAGAGCAACCTAAACAGAGAGGAT      180
MM                  CACTACCAACAGCAGATGGCCCATACCTTCAAATATTAGAGCAACCTAAACAGAGAGGAT      180
                    ************************************************************

NM_001076409.1      TTCGTTTCCGTTACGTGTGTGAAGGCCCCTCCCATGGAGGGCTCCCCGGTGCATCTAGTG      240
HH                  TTCGTTTCCGTTACGTGTGTGAAGGCCCCTCCCATGGAGGGCTCCCCGGTGCATCTAGTG      240
HM                  TTCGTTTCCGTTACGTGTGTGAAGGCCCCTCCCATGGAGGGCTCCCCGGTGCATCTAGTG      240
MH                  TTCGTTTCCGTTACGTGTGTGAAGGCCCCTCCAATGGAGGGCTCCCCGGTGCATCTAGTG      240
MM                  TTCGTTTCCGTTACGTGTGTGAAGGCCCCTCCCATGGAGGGCTCCCCGGTGCATCTAGTG      240
                    ******************************** ***************************

NM_001076409.1      AAAAGAACAAGAAGTCCTACCCTCAGGTCAAAATCTGCAACTATGTGGGACCTGCAAAGG      300
HH                  AAAAGAACAAGAAGTCCTACCCTCAGGTCAAAATCTGCAACTATGTGGGACCTGCAAAGG      300
HM                  AAAAGAACAAGAAGTCCTACCCTCAGGTCAAAATCTGCAACTATGTGGGACCTGCAAAGG      300
MH                  AAAAGAACAAGAAGTCCTACCCTCAGGTCAAAATCTGCAACTATGTGGGACCTGCAAAGG      300
MM                  AAAAGAACAAGAAGTCCTACCCTCAGGTCAAAATCTGCAACTATGTGGGACCTGCAAAGG      300
                    ************************************************************

NM_001076409.1      TTATTGTTCAGTTGGTCACAAATGGAAAAAAACATCCACCTGCATGCACACAGCCTGGTGG      360
HH                  TTATTGTTCAGTTGGTCACAAATGGAAAAAAACATCCACCTGCATGCACACAGCCTGGTGG      360
HM                  TTATTGTTCAGTTGGTCACAAATGGAAAAAAACATCCACCTGCATGCACACAGCCTGGTGG      360
MH                  TTATTGTTCAGTTGGTCACAAATGGAAAAAAACATCCACCTGCATGCACACAGCCTGGTGG      360
MM                  TTATTGTTCAGTTGGTCACAAATGGAAAAAAACATCCACCTGCATGCACACAGCCTGGTGG      360
                    ************************************************************

NM_001076409.1      GAAAACACTGTGAGGATGGCGTCTGCACTGTGACAG  396
HH                  GAAAACACTGTGAGGATGGCGTCTGCACTGTGACAG  396
HM                  GAAAACACTGTGAGGATGGCGTCTGCACTGTGACAG  396
MH                  GAAAACACTGTGAGGATGGCGTCTGCACTGTGACAG  396
MM                  GAAAACACTGTGAGGATGGCGTCTGCACTGTGACAG  396
                    ************************************
```

Figure S2. *NFKB* gene (396-bp) demonstrative DNA sequence alignment between healthy and mastitis affected Holstein and Montbéliarde dairy cows together with the reference sequence found in GenBank gb|NM_001076409.1|. HH= Healthy Holstein; HM= Healthy Montbéliarde; MH= Mastitic Holstein; and MM= Mastitic Montbéliarde.

```
NM_001205541.3      CGTGCAGATCGGCTGGGAGCTGCCGGCGAGAAACTCCCGTGCATCCGCCGGAGCTGCTGG    60
HH                  CGTGCAGATCGGCTGGGAGCTGCCGGCGAGAAACTCCCGTGCATCCGCCGGAGCTGCTGG    60
HM                  CGTGCAGATCGGCTGGGAGCTGCCGGCGAGAAACTCCCGTGCATCCGCCGGAGCTGCTGG    60
MH                  CGTGCAGATCGGCTGGGAGCTGCCGGCGAGAAACTCCCGTGCATCCGCCGGAGCTGCTGG    60
MM                  CGTGCAGATCGGCTGGGAGCTGCCGGCGAGAAACTCCCGTGCATCCGCCGGAGCTGCTGG    60
                    ************************************************************

NM_001205541.3      ACTTCCACGCCCTTCTCCCGCTCCAAGCCAGCTTGAAGGAGCGGTTTCCTCGAAGGAAGG    120
HH                  ACTTCCACGCCCTTCTCCCGCTCCAAGCCAGCTTGAAGGAGCGGTTTCCTCGAAGGAAGG    120
HM                  ACTTCCACGCCCTTCTCCCGCTCCAAGCCAGCTTGAAGGAGCGGTTTCCTCGAAGGTAGG    120
MH                  ACTTCCACGCCCTTCTCCCGCTCCAAGCCAGCTTGAAGGAGCGGTTTCCTCGAAGGAAGG    120
MM                  ACTTCCACGCCCTTCTCCCGCTCCAAGCCAGCTTGAAGGAGCGGTTTCCTCGAAGGAAGG    120
                    ******************************************************** ***

NM_001205541.3      TGGTGTAAACTACAATGACCCTGTGTTAATACCACAGACATCATAGTGGAACTGAAGGGA    180
HH                  TGGTGTAAACTACAATGACCCTGTGTTAATACCACAGACATCATAGTGGAACTGAAGGGA    180
HM                  TGGTGTAAACTACAATGACCCTGTGTTAATACCACAGACATCATAGTGGAACTGAAGGGA    180
MH                  TGGTGTAAACTACAATGACCCTGTGTTAATACCACAGACATCATAGTGGAACTGAAGGGA    180
MM                  TGGTGTAAACTACAATGACCCTGTGTTAATACCACAGACATCATAGTGGAACTGAAGGGA    180
                    ************************************************************

NM_001205541.3      ACTTAATTTACTGGTGTGTTTGTTTGTAACCAGCTTCTGATCGAAAGTAAGAAAAGACAT    240
HH                  ACTTAATTTACTGGTGTGTTTGTTTGTAACCAGCTTCTGATCGAAAGTAAGAAAAGACAT    240
HM                  ACTTAATTTACTGGTGTGTTTGTTTGTAACCAGCTTCTGATCGAAAGTAAGAAAAGACAT    240
MH                  ACTTAATTTACTGGTGTGTTTGTTTGTAACCAGCTTCTGATCGAAAGTAAGAAAAGACAT    240
MM                  ACTTAATTTACTGGTGTGTTTGTTTGTAACCAGCTTCTGATCGAAAGTAAGAAAAGACAT    240
                    ************************************************************

NM_001205541.3      TACGATTTTCATTCTTTCTGGGCTGCCTCCTTCCTGAAGAGCAATGGAGGTGGTGTTAAG    300
HH                  TACGATTTTCATTCTTTCTGGGCTGCCTCCTTCCTGAAGAGCAATGGAGGTGGTGTTAAG    300
HM                  TACGATTTTCATTCTTTCTGGGCTGCCTCCTTCCTGAAGAGCAATGGAGGTGGTGTTAAG    300
MH                  TACGATTTTCATTCTTTCTGGGCTGCCTCCTTCCTGAAGAGCAATGGAGGTGGTGTTAAG    300
MM                  TACGATTTTCATTCTTTCTGGGCTGCCTCCTTCCTGAAGAGCAATGGAGGTGGTGTTAAG    300
                    ************************************************************

NM_001205541.3      TGGAAGAGGATTAATCACATCTCTAATTTTCTTCCTGTTAAAATTCTCAACAGCTATTGA    360
HH                  TGGAAGAGGATTAATCACATCTCTAATTTTCTTCCTGTTAAAATTCTCAACAGCTATTGA    360
HM                  TGGAAGAGGATTAATCACATCTCTAATTTTCTTCCTGTTAAAATTCTCAACAGCTATTGA    360
MH                  TGGAAGAGGATTAATCACATCTCTAATTTTCTTCCTGTTAAAATTCTCAACAGCTATTGA    360
MM                  TGGAAGAGGATTAATCACATCTCTAATTTTCTTCCTGTTAAAATTCTCAACAGCTATTGA    360
                    ************************************************************

NM_001205541.3      TATACCACTTTCAGTTCAACAGGTTCCAACAATCGTGAAGCAGTCAACAGTGCAAGTTGC    420
HH                  TATACCACTTTCAGTTCAACAGGTTCCAACAATCGTGAAGCAGTCAACAGTGCAAGTTGC    420
HM                  TATACCACTTTCAGTTCAACAGGTTCCAACAATCGTGAAGCAGTCAACAGTGCAAGTTGC    420
MH                  TATACCACTTTCAGTTCAACAGGTTCCAACAATCGTGAAGCAGTCAACAGTGCAAGTTGC    420
MM                  TATACCACTTTCAGTTCAACAGGTTCCAACAATCGTGAAGCAGTCAACAGTGCAAGTTGC    420
                    ************************************************************

NM_001205541.3      CTTTCCTTTTGATGAGTATTTTCAAATTGAATGTGTAGCTAAAGGAAATCCAGAACCAAA    480
HH                  CTTTCCTTTTGATGAGTATTTTCAAATTGAATGTGTAGCTAAAGGAAATCCAGAACCAAA    480
HM                  CTTTCCTTTTGATGAGTATTTTCAAATTGAATGTGTAGCTAAAGGAAATCCAGAACCAAA    480
MH                  CTTTCCTTTTGATGAGTATTTTCAAATTGAATGTGTAGCTAAAGGAAATCCAGAACCAAA    480
MM                  CTTTCCTTTTGATGAGTATTTTCAAATTGAATGTGTAGCTAAAGGAAATCCAGAACCAAA    480
                    ************************************************************

NM_001205541.3      ATTCTTGTGGAATAAGGATGACAAGCCTTTTAATCTCTCTGACCCTCGGATAATTGTATC    540
HH                  ATTCTTGTGGAATAAGGATGACAAGCCTTTTAATCTCTCTGACCCTCGGATAATTGTATC    540
HM                  ATTCTTGTGGAATAAGGATGACAAGCCTTTTAATCTCTCTGACCCTCGGATAATTGTATC    540
MH                  ATTCTTGTGGAATAAGGATGACAAGCCTTTTAATCTCTCTGACCCTCGGATAATTGTATC    540
MM                  ATTCTTGTGGAATAAGGATGACAAGCCTTTTAATCTCTCTGACCCTCGGATAATTGTATC    540
                    ************************************************************

NM_001205541.3      TAACAAT  547
HH                  TAACAAT  547
HM                  TAACAAT  547
MH                  TAACAAT  547
MM                  TAACAAT  547
                    *******
```

Figure S3. *CHL1* gene (547-bp) demonstrative DNA sequence alignment between healthy and mastitis affected Holstein and Montbéliarde dairy cows together with the reference sequence found in GenBank gb| NM_001205541.3|. HH= Healthy Holstein; MH= Mastitic Holstein; HM= Healthy Montbéliarde and MM= Mastitic Montbéliarde.

```
NM_001077941.1      CTCTACGCGGCTGTCCGCCTCATTCTCAGAGCAGGTCCGTGAATTGTCGGCGCGCTCCGG      60
HH                  CTCTACGCGGCTGTCCGCCTCATTCTCAGAGCAGGTCCGTGAATTGTCGGCGCGCTCCGG      60
HM                  CTCTACGCGGCTGTCCGCCTCATTCTCAGAGCAGGTCCGTGAATTGTCGGCGCGCTCCGG      60
MH                  CTCTACGCGGCTGTCCGCCTCATTCTCAGAGCAGGTCCGTGAATTGTCGGCGCGCTCCGG      60
MM                  CTCTACGCGGCTGTCCGCCTCATTCTCAGAGCAGGTCCGTGAATTGTCGGCGCGCTCCGG      60
                    ************************************************************

NM_001077941.1      GCAGCTGCTGAGCCGGGGCTACCATCGGGCTTGTCGTGCTCCCTCCGAGGTGCCTTCCGG      120
HH                  GCAGCTGCTGAGCCGGGGCTACCATCGGGCTTGTCGTGCTCCCTCCGAGGTGCCTTCCGG      120
HM                  GCAGCTGCTGAGCCGGGGCTACCATTGGGCTTGTCGTGCTCCCTCCGAGGTGCCTGCCGG      120
MH                  GCAGCTGCTGAGCCGGGGCTACCATCGGGCTTGTCGTGCTCCCTCCGAGGTGCCTTCCGG      120
MM                  GCAGCTGCTGAGCCGGGGCTACCATCGGGCTTGTCGTGCTCCCTCCGAGGTGCCTTCCGG      120
                    ************************ ****************************** ****

NM_001077941.1      AGCTAATGTGCCCCGGTCGGCCAGGGCTGTCGCCGGTTCGGGGCAACTTGGGATTCAGGA      180
HH                  AGCTAATGTGCCCCGGTCGGCCAGGGCTGTCGCCGGTTCGGGGCAACTTGGGATTCAGGA      180
HM                  AGCTAATGTGCCCCGGTCGGCCAGGGCTGTCGCCGGTTCGGGGCAACTTGGGATTCAGGA      180
MH                  AGCTAATGTGCCCCGGTCGGCCAGGGCTGTCGCCGGTTCGGGGCAACTTGGGATTCAGGA      180
MM                  AGCTAATGTGCCCCGGTCGGCCAGGGCTGTCGCCGGTTCGGGGCAACTTGGGATTCAGGA      180
                    ************************************************************

NM_001077941.1      CTCGCAGCAGGGGCGTCGGTTTGCGATCAGGACTCGGCTACGAGGATGGAAATCCTGTAT      240
HH                  CTCGCAGCAGGGGCGTCGGTTTGCGATCAGGACTCGGCTACGAGGATGGAAATCCTGTAT      240
HM                  CTCGCAGCAGGGGCGTCGGTTTGCGATCAGGACTCGGCTACGAGGATGGAAATCCTGTAT      240
MH                  CTCGCAGCAGGGGCGTCGGTTTGCGATCAGGACTCAGCTACGAGGATGGAAATCCTGTAT      240
MM                  CTCGCAGCAGGGGCGTCGGTTTGCGATCAGGACTCAGCTACGAGGATGGAAATCCTGTAT      240
                    *********************************** ************************

NM_001077941.1      GAATGTGGAAGCCATTAGCAGAGTAATTGCTGTCTGTTACCATGACAACCAGCCGCTGCA      300
HH                  GAATGTGGAAGCCATTAGCAGAGTAATTGCTGTCTGTTACCATGACAACCAGCCGCTGCA      300
HM                  GAATGTGGAAGCCATTAGCAGAGTAATTGCTGTCTGTTACCATGACAACCAGCCGCTGCA      300
MH                  GAATGTGGAAGCCATTAGCAGAGTAATTGCTGTCTGTTACCATGACAACCAGCCGCTGCA      300
MM                  GAATGTGGAAGCCATTAGCAGAGTAATTGCTGTCTGTTACCATGACAACCAGCCGCTGCA      300
                    ************************************************************

NM_001077941.1      GTCACCTGCCCGAAGTGCTACCAGACTGCACCGGCTCCGCTGCACCCGTGGTGAAGACCG      360
HH                  GTCACCTGCCCGAAGTGCTACCAGACTGCACCGGCTCCGCTGCACCCGTGGTGAAGACCG      360
HM                  GTCACCTGCCCGAAGTGCTACCAGACTGCACCGGCTCCGCTGCACCCGTGGTGAAGACCG      360
MH                  GTCACCTGCCCGAAGTGCTACCAGACTGCACCGGCTCCGCTGCACCCGTGGTGAAGACCG      360
MM                  GTCACCTGCCCGAAGTGCTACCAGACTGCACCGGCTCCGCTGCACCCGTGGTGAAGACCG      360
                    ************************************************************

NM_001077941.1      TGGAGGACTGTGGCAGCCTGGTGAATGGGCAGCCGCAGTATGTCATGCAAGTTTCCGCTA      420
HH                  TGGAGGACTGTGGCAGCCTGGTGAATGGGCAGCCGCAGTATGTCATGCAAGTTTCCGCTA      420
HM                  TGGAGGACTGTGGCAGCCTGGTGAATGGGCAGCCGCAGTATGTCATGCAAGTTTCCGCTA      420
MH                  TGGAGGACTGTGGCAGCCTGGTGAATGGGCAGCCGCAGTATGTCATGCAAGTTTCCGCTA      420
MM                  TGGAGGACTGTGGCAGCCTGGTGAATGGGCAGCCGCAGTATGTCATGCAAGTTTCCGCTA      420
                    ************************************************************

NM_001077941.1      AGGACGGGCAGCTGCTGTCAACAGTAGTGCGGACC   455
HH                  AGGACGGGCAGCTGCTGTCAACAGTAGTGCGGACC   455
HM                  AGGACGGGCAGCTGCTGTCAACAGTAGTGCGGACC   455
MH                  AGGACGGGCAGCTGCTGTCAACAGTAGTGCGGACC   455
MM                  AGGACGGGCAGCTGCTGTCAACAGTAGTGCGGACC   455
                    ***********************************
```

Figure S4. *MARCHF3* gene (455-bp) demonstrative DNA sequence alignment between healthy and mastitis affected Holstein and Montbéliarde dairy cows together with the reference sequence found in GenBank gb| NM_001077941.1|. HH= Healthy Holstein; MH= Mastitic Holstein; HM= Healthy Montbéliarde and MM= Mastitic Montbéliarde.

```
NM_001083706.1      GCCAGCGAGTGCGGGCGCGCGTCCCGGGAGCCTGTCCCGGCTTTTTCTTGGAGTGACGCT      60
HH                  GCCAGCGAGTGCGGGCGCGCGTCCCGGGAGCCTGTCCCGGCTTTTTCTTGGAGTGACGCT      60
HM                  GCCAGCGAGTGCGGGCGCGCGTCCCGGGAGCCTGTCCCGGCTTTTTCTTGGAGTGACGCT      60
MH                  GCCAGCGAGTGCGGGCGCGCGTCCCGGGAGCCTGTCCCGGCTTTTTCTTGGAGTGACGCT      60
MM                  GCCAGCGAGTGCGGGCGCGCGTCCCGGGAGCCTGTCCCGGCTTTTTCTTGGAGTGACGCT      60
                    ************************************************************

NM_001083706.1      GTCCCCGGGTCTCTGATCCCAATGCACCGGCTCGTCCTTGTCTATACGCTAGTCTGCGCA      120
HH                  GTCCCCGGGTCTCTGATCCCAATGCACCGGCTCGTCCTTGTCTATACGCTAGTCTGCGCA      120
HM                  GTCCCCGGGTCTCTGATCCCAATGCACCGGCTCGTCCTTGTCTATACGCTAGTCTGCGCA      120
MH                  GTCCCCGGGTCTCTGATCCCAATGCACCGGCTCATCCTTGTCTATACGCTAGTCTGCGCA      120
MM                  GTCCCCGGGTCTCTGATCCCAATGCACCGGCTCGTCCTTGTCTATACGCTAGTCTGCGCA      120
                    ********************************* **************************

NM_001083706.1      AACTTTTGCAGCTACCGGGACACTTCTGCCACCCCGCAGAGCGCATCTATCAAAGCTTTG      180
HH                  AACTTTTGCAGCTACCGGGACACTTCTGCCACCCCGCAGAGCGCATCTATCAAAGCTTTG      180
HM                  AACTTTTGCAGCTACCGGGGCACTTCTGCCACCCCGCAGAGCGCATCTATCAAAGCTTTG      180
MH                  AACTTTTGCAGCTACCGGGACACTTCTGCCACCCCGCAGAGCGCATCTATCAAAGCTTTG      180
MM                  AACTTTTGCAGCTACCGGGACACTTCTGCCACCCCGCAGAGCGCATCTATCAAAGCTTTG      180
                    ****************** *****************************************

NM_001083706.1      CGTAACGCCAACCTCAGGCGAGATGACTTGTACCGAAGAGACGAGACCATCGAGGTGACA      240
HH                  CGTAACGCCAACCTCAGGCGAGATGACTTGTACCGAAGAGACGAGACCATCCAGGTGACA      240
HM                  CGTAACGCCAACCTCAGGCGAGATGACTTGTACCGAAGAGACGAGACCATCGAGGTGACA      240
MH                  CGTAACGCCAACCTCAGGCGAGATGACTTGTACCGAAGAGACGAGACCATCGAGGTGACA      240
MM                  CGTAACGCCAACCTCAGGCGAGATGACTTGTACCGAAGAGACGAGACCATCGAGGTGACA      240
                    *************************************************** ********

NM_001083706.1      GGACATGGCCACGTGCAGAGTCCCCGCTTCCCAAACAGCTACCCTCGCAACCTGCTTCTG      300
HH                  GGACATGGCCACGTGCAGAGTCCCCGCTTCCCAAACAGCTACCCTCGCAACCTGCTTCTG      300
HM                  GGACATGGCCACGTGCAGAGTCCCCGCTTCCCAAACAGCTACCCTCGCAACCTGCTTCTG      300
MH                  GGACATGGCCACGTGCAGAGTCCCCGCTTCCCAAACAGCTACCCTCGCAACCTGCTTCTG      300
MM                  GGACATGGCCACGTGCAGAGTCCCCGCTTCCCAAACAGCTACCCTCGCAACCTGCTTCTG      300
                    ************************************************************

NM_001083706.1      ACCTGGCGGCTCCACTCCCAGGAGAAAACAAGGATACAGCTAGCCTTTGACAATCAGTTT      360
HH                  ACATGGCGGCTCCACTCCCAGGAGAAAACAAGGATACAGCTAGCCTTTGACAATCAGTTT      360
HM                  ACATGGCGGCTCCACTCCCAGGAGAAAACAAGGATACAGCTAGCCTTTGACAATCAGTTT      360
MH                  ACCTGGCGGCTCCACTCCCAGGAGAAAACAAGGATACAGCTAGCCTTTGACAATCAGTTT      360
MM                  ACCTGGCGGCTCCACTCCCAGGAGAAAACAAGGATACAGCTAGCCTTTGACAATCAGTTT      360
                    ** *********************************************************

NM_001083706.1      GGATTAGAGGAAGCCGAAAATGATATCTGTAGGTATGATTTTGTAGAAGTTGAAGACATA      420
HH                  GGATTAGAGGAAGCCGAAAATGATATCTGTAGGTATGATTTTGTAGAAGTTGAAGACATA      420
HM                  GGATTAGAGGAAGCCGAAAATGATATCTGTAGGTATGATTTTGTAGAAGTTGAAGACATA      420
MH                  GGATTAGAGGAAGCCGAAAATGATATCTGTAGGTATGATTTTGTAGAAGTTGAAGACATA      420
MM                  GGATTAGAGGAAGCCGAAAATGATATCTGTAGGTATGATTTTGTAGAAGTTGAAGACATA      420
                    ************************************************************

NM_001083706.1      TCTGAAACCAGTACTGTTATTAGAGGACGATGGTGTGGACACAAGGAAGTTCCTCCAAGG      480
HH                  TCTGAAACCAGTACTGTTATTAGAGGACGATGGTGTGGACACAAGGAAGTTCCTCCAAGG      480
HM                  TCTGAAACCAGTACTGTTATTAGAGGACGATGGTGTGGACACAAGGAAGTTCCTCCAAGG      480
MH                  TCTGAAACCAGTACTGTTATTAGAGGACGATGGTGTGGACACAAGGAAGTTCCTCCAAGG      480
MM                  TCTGAAACCAGTACTGTTATTAGAGGACGATGGTGTGGACACAAGGAAGTTCCTCCAAGG      480
                    ************************************************************

NM_001083706.1      ATAATATCAAGAACAAACCAGATTAAAATAACGTTCAAGTCTGATGACTAC      531
HH                  ATAATATCAAGAACAAACCAGATTAAAATAACGTTCAAGTCTGATGACTAC      531
HM                  ATAATATCAAGAACAAACCAGATTAAAATAACGTTCAAGTCTGATGACTAC      531
MH                  ATAATATCAAGAACAAACCAGATTAAAATAACGTTCAAGTCTGATGACTAC      531
MM                  ATAATATCAAGAACAAACCAGATTAAAATAACGTTCAAGTCTGATGACTAC      531
                    ***************************************************
```

Figure S5. *PDGFD* gene (531-bp) demonstrative DNA sequence alignment between healthy and mastitis affected Holstein and Montbéliarde dairy cows together with the reference sequence found in GenBank gb|NM_001083706.1|. HH= Healthy Holstein; MH= Mastitic Holstein; HM= Healthy Montbéliarde and MM= Mastitic Montbéliarde.

```
XM_024994781.1      TCCTGTTACCGCTCCTTACCCAGACTGGAGTGTCTGTCCGCCAGGGTCAGCTCAGGTGGT      60
HH                  TCCTGTTACCGCTCCTTACCCAGACTGGAGTGTCTGTCCGCCAGGGTCAGCTCAGGTGGT      60
HM                  TCCTGTTACCGCTCCTTACCCAGACTGGAGTGTCTGTCCGCCAGGGCCAGCTCAGGTGGT      60
MH                  TCCTGTTACCGCTCCTTACCCAGACTGGAGTGTCTGTCCGCCAGGGTCAGCTCAGGTGGT      60
MM                  TCCTGTTACCGCTCCTTACCCAGACTGGAGTGTCTGTCCGCCAGGGTCAGCTCAGGTGGT      60
                    ********************************************** *************

XM_024994781.1      GACAGAGGCTGTAGGGATTTGGGACTGAATCAGGATTCGAACCTAGGTCAGTCCTCTGGC      120
HH                  GACAGAGGCTGTAGGGATTTGGGACTGAATCAGGATTCGAACCTAGGTCAGTCCTCTGGC      120
HM                  GACAGAGGCTGTAGGGATTTGGGACTGAATCAGGATTCGAACCTAGGTCAGTCCTCTGGC      120
MH                  GACAGAGGCTGTAGGGATTTGGGACTGAATCAGGATTCGAACCTAGGTCAGTCCTCTGGC      120
MM                  GACAGAGGCTGTAGGGATTTGGGACTGAATCAGGATTCGAACCTAGGTCAGTCCTCTGGC      120
                    ************************************************************

XM_024994781.1      CAGACCTGGGGAGGAGCTTCTTGGAAAAACTGAGACTCTTTGGTCCTTGGGGCCCAGTCA      180
HH                  CAGACCTGGGGAGGAGCTTCTTGGAAAAACTGAGACTCTTTGGTCCTTGGGGCCCAGTCA      180
HM                  CAGACCTGGGGAGGAGCTTCTTGGAAAAACTGAGGCTCTTTGGTCCTTGGGGCCCAGTCA      180
MH                  CAGACCTGGGGAGGAGCTTCTTGGAAAAACTGAGACTCTTTGGTCCTTGGGGCCCAGTCA      180
MM                  CAGACCTGGGGAGGAGCTTCTTGGAAAAACTGAGACTCTTTGGTCCTTGGGGCCCAGTCA      180
                    ********************************** *************************

XM_024994781.1      GCGGAAATGTTGGTTCAGAAGTAAACATTTGCTGCAGGAGGCTGGTGTGCATGTCGGTTA      240
HH                  GCGGAAATGTTGGTTCAGAAGTAAACATTTGCTGCAGGAGGCTGGTGTGCATGTCGGTTA      240
HM                  GCGGAAATGTTGGTTCAGAAGTAAACATTTGCTGCAGGAGGCTGGTGTGCATGTCGGTTA      240
MH                  GCGGAAATGTTGGTTCAGAAGTAAACATTTGCTGCAGGAGGCTGGTGTGCATGTCGGTTA      240
MM                  GCGGAAATGTTGGTTCAGAAGTAAACATTTGCTGCAGGAGGCTGGTGTGCATGTCGGTTA      240
                    ************************************************************

XM_024994781.1      TCAGGCGGGGGTGAAAAATCACTTCGTTCTATACAGAGCAAAGTGGAGTCTCAGTTTCTC      300
HH                  TCAGGCGGGGGTGAAAAATCACTTCGTTCTATACAGAGCAAAGTGGAGTCTCAGTTTCTC      300
HM                  TCAGGCGGGGGTGAAAAATCACTTCGTTCTATACAGAGCAAAGTGGAGTCTCAGTTTCTC      300
MH                  TCAGGCGGGGGTGAAAAATCACTTCGTTCTATACAGAGCAAAGTGGAGTCTCAGTTTCTC      300
MM                  TCAGGCGGGGGTGAAAAATCACTTCGTTCTATACAGAGCAAAGTGGAGTCTCAGTTTCTC      300
                    ************************************************************

XM_024994781.1      CTTCTGAAAAGCAGAGAAGCTAAGTGAGGGCTGTGAGCCCTGCTGGACCTATGTGGAAAT      360
HH                  CTTCTGAAAAGCAGAGAAGCTAAGTGAGGGCTGTGAGCCCTGCTGGACCTATGTGGAAAT      360
HM                  CTTCTGAAAAGCAGAGAAGCTAAGTGAGGGCTGTGAGCCCTGCTGGACCTATGTGGAAAT      360
MH                  CTTCTGAAAAGCAGAGAAGCTAAGTGAGGGCTGTGAGCCCTGCTGGACCTATGTGGAAAT      360
MM                  CTTCTGAAAAGCAGAGAAGCTAAGTGAGGGCTGTGAGCCCTGCTGGACCTATGTGGAAAT      360
                    ************************************************************

XM_024994781.1      TCCAGCTCAGCCAATGTCAGGATATGTGACCTCAGGCAGTTCCTTAACCTCTCTGTGCTT      420
HH                  TCCAGCTCAGCCAATGTCAGGATCTGTGACCTCAGGCAGTTCCTTAACCTCTCTGTGCTT      420
HM                  TCCAGCTCAGCCAATGTCAGGATATGTGACCTCAGGCAGTTCCTTAACCTCTCTGTGCTT      420
MH                  TCCAGCTCAGCCAATGTCAGGATATGTGACCTCAGGCAGTTCCTTAACCTCTCTGTGCTT      420
MM                  TCCAGCTCAGCCAATGTCAGGATATGTGACCTCAGGCAGTTCCTTAACCTCTCTGTGCTT      420
                    *********************** ************************************

XM_024994781.1      AAGTGTCTCACCAGTGCTTGGTCTAGGCATTCCCAGAGGGAACACAGGCAAAGGCTGAGT      480
HH                  AAGTGTCTCACCAGTGCTTGGTCTAGGCATTCCCAGAGGGAACACAGGCAAAGGCTGAGT      480
HM                  AAGTGTCTCACCAGTGCTTGGTCTAGGCATTCCCAGAGGGAACACAGGCAAAGGCTGAGT      480
MH                  AAGTGTCTCACCAGTGCTTGGTCTAGGCATTCCCAGAGGGAACACAGGCAAAGGCTGAGT      480
MM                  AAGTGTCTCACCAGTGCTTGGTCTAGGCATTCCCAGAGGGAACACAGGCAAAGGCTGAGT      480
                    ************************************************************

XM_024994781.1      AATGGGACCAGCAGCTGAAGGACCTTGAATGCCTAGTTTAGGATTCAGGCTCTTCTTCCA      540
HH                  AATGGGACCAGCAGCTGAAGGACCTTGAATGCCTAGTTTAGGATTCAGGCTCTTCTTCCA      540
HM                  AATGGGACCAGCAGCTGAAGGACCTTGAATGCCTAGTTTAGGATTCAGGCTCTTCTTCCA      540
MH                  AATGGGACCAGCAGCTGAAGGACCTTGAATGCCTAGTTTAGGATTCAGGCTCTTCTTCCA      540
MM                  AATGGGACCAGCAGCTGAAGGACCTTGAATGCCTAGTTTAGGATTCAGGCTCTTCTTCCA      540
                    ************************************************************

XM_024994781.1      GAACCCGGGGAGCCGCAGAAGGCTCATGGGCCCCTCAGGGATCAGGTGCCAGAAACTGAC      600
HH                  GAACCCGGGGAGCCGCAGAAGGCTCATGGGCCCCTCAGGGATCAGGTGCCAGAAACTGAC      600
HM                  GAACCCGGGGAGCCGCAGAAGGCTCATGGGCCCCTCAGGGATCAGGTGCCAGAAACTGAC      600
MH                  GAACCCGGGGAGCCGCAGAAGGCTCATGGGCCCCTCAGGGATCAGGTGCCAGAAACTGAC      600
MM                  GAACCCGGGGAGCCGCAGAAGGCTCATGGGCCCCTCAGGGATCAGGTGCCAGAAACTGAC      600
                    ************************************************************

XM_024994781.1      CTCAGGCCCCTCTTTTCTAACATACTTCTCACTCTCTGTTGAACCACAAG      650
HH                  CTCAGGCCCCTCTTTTCTAACATACTTCTCACTCTCTGTTGAACCACAAG      650
HM                  CTCAGGCCCCTCTTTTCTAACATACTTCTCACTCTCTGTTGAACCACAAG      650
MH                  CTCAGGCCCCTCTTTTCTAACATACTTCTCACTCTCTGTTGAACCACAAG      650
MM                  CTCAGGCCCCTCTTTTCTAACATACTTCTCACTCTCTGTTGAACCACAAG      650
                    **************************************************
```

Figure S6. *MAST3* gene (650-bp) demonstrative DNA sequence alignment between healthy and mastitis affected Holstein and Montbéliarde dairy cows together with the reference sequence found in GenBank gb| XM_024994781.1|. HH= Healthy Holstein; MH= Mastitic Holstein; HM= Healthy Montbéliarde and MM= Mastitic Montbéliarde.

```
XM_024993963.1      TCCATTATATGAGTCTTACTACAAGCAGGTAGACCCAGCATACACAGGGCGAGTTGGGGC     60
HH                  TCCATTATATGAGTCTTACTACAAGCAGGTAGATCCAGCATACACAGGGCGAGTTGGGGC     60
HM                  TCCATTATATGAGTCTTACTACAAGCAGGTAGATCCAGCATACACAGGGCGAGTTGGGGC     60
MH                  TCCATTATATGAGTCTTACTACAAGCAGGTAGACCCAGCATACACAGGGCGAGTTGGGGC     60
MM                  TCCATTATATGAGTCTTACTACAAGCAGGTAGACCCAGCATACACAGGGCGAGTTGGGGC     60
                    ********************************* **************************

XM_024993963.1      GAGTGAAGCTGCACTTTTTCTAAAGAAGTCTGGACTCTCAGACATTATCCTTGGGAAGAT     120
HH                  GAGTGAAGCTGCACTTTTTCTAAAGAAGTCTGGACTCTCAGACATTATCCTTGGGAAGAT     120
HM                  GAGTGAAGCTGCACTTTTTCTAAAGAAGTCTGGACTCTCAGACATTATCCTTGGGAAGAT     120
MH                  GAGTGAAGCTGCACTTTTCCTAAAGAAGTCTGGACTCTCAGACATTATCCTTGGGAAGAT     120
MM                  GAGTGAAGCTGCACTTTTTCTAAAGAAGTCTGGACTCTCAGACATTATCCTTGGGAAGAT     120
                    ****************** *****************************************

XM_024993963.1      ATGGGACTTGGCTGACCCAGAAGGTAAAGGGTACTTGGACAAACAGGGTTTCTATGTTGC     180
HH                  ATGGGACTTGGCTGACCCAGAAGGTAAAGGGTACTTGGACAAACAGGGTTTCTATGTTGC     180
HM                  ATGGGACTTGGCTGACCCAGAAGGTAAAGGGTACTTGGACAAACAGGGTTTCTATGTTGC     180
MH                  ATGGGACTTGGCTGACCCAGAAGGTAAAGGGTACTTGGACAAACAGGGTTTCTATGTTGC     180
MM                  ATGGGACTTGGCTGACCCAGAAGGTAAAGGGTACTTGGACAAACAGGGTTTCTATGTTGC     180
                    ************************************************************

XM_024993963.1      ACTGAGACTAGTCGCCTGCGCACAGAGCGGCCACGAGGTTACCTTGAGCAATCTGAATTT     240
HH                  ACTGAGACTAGTCGCCTGCGCACAGAGCGGCCACGAGGTTACCTTGAGCAATCTGAATTT     240
HM                  ACTGAGACTAGTCGCCTGCGCGCAGAGCGGCCACGAGGTTACCTTGAGCAATCTGAATTT     240
MH                  ACTGAGACTAGTCGCCTGCGCACAGAGCGGCCACGAGGTTACCTTGAGCAATCTGAATTT     240
MM                  ACTGAGACTAGTCGCCTGCGCACAGAGCGGCCACGAGGTTACCTTGAGCAATCTGAATTT     240
                    ******************** ***************************************

XM_024993963.1      GAACATGCCACCGCCTAAATTTCACGACAGCAGCAGCCCTCTCATGGTCACGGCGCCTTC     300
HH                  GAACATGCCACCGCCTAAATTTCACGACAGCAGCAGCCCTCTCATGGTCACGGCGCCTTC     300
HM                  GAACATGCCACCGCCTAAATTTCACGACAGCAGCAGCCCGCTCATGGTCACGGCGCCTTC     300
MH                  GAACATGCCGCCGCCTAAATTTCACGACAGCAGCAGCCCTCTCATGGTCACGGCGCCTTC     300
MM                  GAACATGCCACCGCCTAAATTTCACGACAGCAGCAGCCCTCTCATGGTCACGGCGCCTTC     300
                    ********* ***************************** ********************

XM_024993963.1      TGCAGAGGCCCACTGGGCTGTGAGGGTAGAAGAAAAGGCAAAATTTGATGGAATTTTTGA     360
HH                  TGCAGAGGCCCACTGGGCTGTGAGGGTAGAAGAAAAGGCAAAATTTGATGGAATTTTTGA     360
HM                  TGCAGAGGCCCACTGGGCTGTGAGGGTAGAAGAAAAGGCAAAATTTGATGGAATTTTTGA     360
MH                  TGCAGAGGCCCACTGGGCTGTGAGGGTAGAAGAAAAGGCAAAATTTGATGGAATTTTTGA     360
MM                  TGCAGAGGCCCACTGGGCTGTGAGGGTAGAAGAAAAGGCAAAATTTGATGGAATTTTTGA     360
                    ************************************************************

XM_024993963.1      AAGCCTCTTACCTGTCAATGGTT     383
HH                  AAGCCTCTTACCTGTCAATGGTT     383
HM                  AAGCCTCTTACCTGTCAATGGTT     383
MH                  AAGCCTCTTACCTGTCAATGGTT     383
MM                  AAGCCTCTTACCTGTCAATGGTT     383
                    ***********************
```

Figure S7. *EPS15L1* gene (383-bp) demonstrative DNA sequence alignment between healthy and mastitis affected Holstein and Montbéliarde dairy cows together with the reference sequence found in GenBank gb|XM_024993963.1|. HH= Healthy Holstein; MH= Mastitic Holstein; HM= Healthy Montbéliarde and MM= Mastitic Montbéliarde.

```
NM_001101138.1      CGAGGAGACCACGGCGGCCAGAGCCCCAGCCGTCTTCCCCGGGAAGCTCTGTGGCTCTGT      60
HH                  CGAGGAGACCACGGCGGCCAGAGCCCCAGCCGTCTTCCCCGGGAAGCTCTGTGGCTCTGT      60
HM                  CGAGGAGACCACGGCGGCCAGAGCCCCAGCCGTCTTCCCCGGGAAGCTCTGTGGCTCTGT      60
MH                  CGAGGAGACCACGGCGGCCAGAGCCCCAGCCGTCTTCCCCGGGAAGCTCTGTGGCTCTGT      60
MM                  CGAGGAGACCACGGCGGCCAGAGCCCCAGCCGTCTTCCCCAGGAAGCTCTGTGGCTCTGT      60
                    **************************************** *******************

NM_001101138.1      TGAGAACCATGCTGGGGAGGCAGCTCGTCTATTGGCACCTTCTGGCTTTGCTTTTCCTCC      120
HH                  TGAGAACCATGCTGGGGAGGCAGCTCGTCTATTGGCACCTTCTGGCTTTGCTTTTCCTCC      120
HM                  TGAGAACCATGCTGGGGAGGCAGCTCGTCTATTGGCACCTTCTGGCTTTGCTTTTCCTCC      120
MH                  TGAGAACCATGCTGGGGAGGCAGCTCGTCTATTGGCACCTTCTGGCTTTGCTTTTCCTCC      120
MM                  TGAGAACCATGCTGGGGAGGCAGCTCGTCTATTGGCACCTTCTGGCTTTGCTTTTCCTCC      120
                    ************************************************************

NM_001101138.1      CTTTTTGCCTGTGTCAAGATGAATACATGGAGTCTCCACAAACCGGAGGACTGCCCCCAG      180
HH                  CTTTTTGCCTGTGTCAAGATGAATACATGGAGTCTCCACAAACCGGAGGACTGCCCCCAG      180
HM                  CTTTTTGCCTGTGTCAAGATGAATACATGGAGTCTCCACAAACCGGAGGACTGCCCCCAG      180
MH                  CTTTTTGCCTGTGTCAAGATGAATACATGGAGTCTCCACAAACCGGAGGACTGCCCCCAG      180
MM                  CTTTTTGCCTGTGTCAAGATGAATACATGGAGTCTCCACAAACCGGAGGACTGCCCCCAG      180
                    ************************************************************

NM_001101138.1      ACTGCAGCAAGTGTTGCCATGGAGACTACAGCTTCCGAGGCTACCAAGGACCCCCTGGAC      240
HH                  ACTGCAGCAAGTGTTGCCATGGAGACTACAGCTTCCGAGGCTACCAAGGACCCCCTGGAC      240
HM                  ACTGCAGCAAGTGTTGCCATGGAGACTACAGCTTCCGAGGCTACCAAGGACCCCCTGGAC      240
MH                  ACTGCAGCAAGTGTTGCCATGGAGACTACAGCTTCCGAGGCTACCAAGGACCCCCTGGAC      240
MM                  ACTGCAGCAAGTGTTGCCATGGAGACTACAGCTTCCGAGGCTACCAAGGACCCCCTGGAC      240
                    ************************************************************

NM_001101138.1      CTCCCGGTCCCCCTGGCATTCCAGGAAACCATGGGAACAATGGCAATAATGGAGCCACTG      300
HH                  CTCCCGGTCCCCCTGGCATTCCAGGAAACCATGGGAACAATGGCAATAATGGAGCCACTG      300
HM                  CTCCCGGTCCCCCTGGCATTCCAGGAAACCATGGGAACAATGGCAATAATGGAGCCACTG      300
MH                  CTCCCGGTCCCCCTGGCATTCCAGGAAACCATGGGAACAATGGCAATAATGGAGCCACTG      300
MM                  CTCCCGGTCCCCCTGGCATTCCAGGAAACCATGGGAACAATGGCAATAATGGAGCCACTG      300
                    ************************************************************

NM_001101138.1      GCCACGAAGGGGCCAAAGGTGAGAAAGGAGACAAAGGCGACCTGGGACCACGAGGGGAGC      360
HH                  GCCACGAAGGGGCCAAAGGTGAGAAAGGAGACAAAGGCGACCTGGGACCACGAGGGGAGC      360
HM                  GCCACGAAGGGGCCAAAGGTGAGAAAGGAGACAAAGGCGACCTGGGACCACGAGGGGAGC      360
MH                  GCCACGAAGGGGCCAAAGGTGAGAAAGGAGACAAAGGCGACCTGGGACCACGAGGGGAGC      360
MM                  GCCACGAAGGGGCCAAAGGTGAGAAAGGAGACAAAGGCGACCTGGGACCACGAGGGGAGC      360
                    ************************************************************

NM_001101138.1      GTGGGCAGCATGGCCCCAAAGGAGAGAAGGGCTACCCGGGGATTCCACCAGAACTGCAGA      420
HH                  GTGGGCAGCATGGCCCCAAAGGAGAGAAGGGCTACCCGGGGATTCCACCAGAACTGCAGA      420
HM                  GTGGGCAGCATGGCCCCAAAGGAGAGAAGGGCTACCCGGGGATTCCACCAGAACTGCAGA      420
MH                  GTGGGCAGCATGGCCCCAAAGGAGAGAAGGGCTACCCGGGGATTCCACCAGAACTGCAGA      420
MM                  GTGGGCAGCATGGCCCCAAAGGAGAGAAGGGCTACCCGGGGATTCCACCAGAACTGCAGA      420
                    ************************************************************

NM_001101138.1      TTGCGTTCATGGCTTCCCTGGCAACTCACTTCACCAATCAGAACAGTGGGATCATTTTCA      480
HH                  TTGCGTTCATGGCTTCCCTGGCAACTCACTTCACCAATCAGAACAGTGGGATCATTTTCA      480
HM                  TTGCGTTCATGGCTTCCCTGGCAACTCACTTCACCAATCAGAACAGTGGGATCATTTTCA      480
MH                  TTGCGTTCATGGCTTCCCTGGCAACTCACTTCACCAATCAGAACAGTGGGATCATTTTCA      480
MM                  TTGCGTTCATGGCTTCCCTGGCAACTCACTTCACCAATCAGAACAGTGGGATCATTTTCA      480
                    ************************************************************

NM_001101138.1      GCAGTGTTGAAACCAACATTGGAAACTTCTTTGATGTCATGACCGG        526
HH                  GCAGTGTTGAAACCAACATTGGAAACTTCTTTGATGTCATGACCGG        526
HM                  GCAGTGTTGAAACCAACATTGGAAACTTCTTTGATGTCATGACCGG        526
MH                  GCAGTGTTGAAACCAACATTGGAAACTTCTTTGATGTCATGACCGG        526
MM                  GCAGTGTTGAAACCAACATTGGAAACTTCTTTGATGTCATGACCGG        526
                    **********************************************
```

Figure S8. *C1QTNF3* gene (526-bp) demonstrative DNA sequence alignment between healthy and mastitis affected Holstein and Montbéliarde dairy cows together with the reference sequence found in GenBank gb| NM_001101138.1|. HH= Healthy Holstein; MH= Mastitic Holstein; HM= Healthy Montbéliarde and MM= Mastitic Montbéliarde.

```
NM_001242563.2      CCGCTGAAGGCGCCGCTCCGCCGCCCCGAAAGACTGGCTTCTTCTGGGCGCTTCGCCTGG      60
HH                  CCGCTGAAGGCGCCGCTCCGCCGCCCTGAAAGACTGGCTTCTTCTGGGCGCTTCGCCTGG      60
HM                  CCGCTGAAGGCGCCGCTCCGCCGCCCCGAAAGACTGGCTTCTTCTGGGCGCTTCGCCTGG      60
MH                  CCGCTGAAGGCGCCGCTCCGCCGCCCCGAAAGACTGGCTTCTTCTGGGCGCTTCGCCTGG      60
MM                  CCGCTGAAGGCGCCGCTCCGCCGCCCCGAAAGACTGGCTTCTTCTGGGCGCTTCGCCTGG      60
                    ************************** *********************************

NM_001242563.2      GTGCTTCTGCTGGCGCCGCTGCTCCTGCTGCCCACGTCCTCCGATGCCTGTGATGATCCA      120
HH                  GTGCTTCTGCTGGCGCCGCTGCTCCTGCTGCCCACGTCCTCCGATGCCTGTGATGATCCA      120
HM                  GTGCTTCTGCTGGCGCCGCTGCTCCTGCTGCCCACGTCCTCCGATGCCTGTGATGATCCA      120
MH                  GTGCTTCTGCTGGCGCCGCTGCTCCTGCTGCCCACGTCCTCCGATGCCTGTGATGATCCA      120
MM                  GTGCTTCTGCTGGCGCCGCTGCTCCTGCTGCCCACGTCCTCCGATGCCTGTGATGATCCA      120
                    ************************************************************

NM_001242563.2      CCAAGATTTGTCTCTATGAAGCCCCAGGGTACCCTTAAACCCAGTTATAGTCCTGGGGAG      180
HH                  CCAAGATTTGTCTCTATGAAGCCCCAGGGTACCCTTAAACCCAGTTATAGTCCTGGGGAG      180
HM                  CCAAGATTTGTCTCTATGAAGCCCCAGGGTACCCTTAAACCCAGTTATAGTCCTGGGGAG      180
MH                  CCAAGATTTGTCTCTATGAAGCCCCAGGGTACCCTTAAACCCAGTTATAGTCCTGGGGAG      180
MM                  CCAAGATTTGTCTCTATGAAGCCCCAGGGTACCCTTAAACCCAGTTATAGTCCTGGGGAG      180
                    ************************************************************

NM_001242563.2      CAGATTGTGTATGAATGTCGTCTGGGTTTCCAGCCAGTAACTCCTGGTCAAGTCCTGGCT      240
HH                  CAGATTGTGTATGAATGTCGTCTGGGTTTCCAGCCAGTAACTCCTGGTCAAGTCCTGGCT      240
HM                  CAGATTGTGTATGAATGTCGTCTGGGTTTCCAGCCAGTAACTCCTGGTCAAGTCCTGGCT      240
MH                  CAGATTGTGTATGAATGTCGTCTGGGTTTCCAGCCAATAACTCCTGGTCAAGTCCTGGCT      240
MM                  CAGATTGTGTATGAATGTCGTCTGGGTTTCCAGCCAGTAACTCCTGGTCAAGTCCTGGCT      240
                    ************************************ ***********************

NM_001242563.2      CTCGTTTGTCAGGATAATAATACATGGTCGTCTCTCCAGGAGGGCTGT         288
HH                  CTTGTTTGTCAGGATAATAATACATGGTCGTCTCTCCAGGAGGGCTGT         288
HM                  CTTGTTTGTCAGGATAATAATACATGGTCGTCTCTCCAGGAGGGCTGT         288
MH                  CTCGTTTGTCAGGATAATAATACATGGTCGTCTCTCCAGGAGGGCTGT         288
MM                  CTCGTTTGTCAGGATAATAATACATGGTCGTCTCTCCAGGAGGGCTGT         288
                    ** *********************************************
```

Figure S9. *CD46* gene (288-bp) demonstrative DNA sequence alignment between healthy and mastitis affected Holstein and Montbéliarde dairy cows together with the reference sequence found in GenBank gb| NM_001242563.2|. HH= Healthy Holstein; MH= Mastitic Holstein; HM= Healthy Montbéliarde and MM= Mastitic Montbéliarde.

```
NM_001082437.2      TGCGAGCGCGCGTGGTCTGTGAGGCCGGCGTTTCGGACCCAACTCTGGCGCGGAGATGCT      60
HH                  TGCGAGCGCGCGTGGTCTGTGAGGCCGGCGTTTCGGACCCAACTCTGGCGCGGAGATGCT      60
HM                  TGCGAGCGCGCGTGGTCTGTGAGGCCGGCGTTTCGGACCCAACTCTGGCGCGGAGATGCT      60
MH                  TGCGAGCGCGCGTGGTCTGTGAGGCCGGCGTTTCGGACCCAACTCTGGCGCGGAGATGCT      60
MM                  TGCGAGCGCGCGTGGTCTGTGAGGCCGGCGTTTCGGACCCAACTCTGGCGCGGAGATGCT      60
                    ************************************************************

NM_001082437.2      GTGCCGGCTCAGCGTTAGGTGGCTGCGGCCCCGGCCTGCCCTGCAGGTCCGTGTCCTGGA      120
HH                  GTGCCGGCTCAGCGTTAGGTGGCTGCGGCCCCGGCCTGCCCTGCAGGTCCGTGTCCTGGA      120
HM                  GTGCCGGCTCAGCGTTAGGTGGCTGCGGCCCCGGCCTGCCCTGCAGGTCCGTGTCCTGGA      120
MH                  GTGCCGGCTCAGCGTTAGGTGGCTGCGGCCCCGGCCTGCCCTGCAGGTCCGTGTCCTGGA      120
MM                  GTGCCGGCTCAGCGTTAGGTGGCTGCGGCCCCGGCCTGCCCTGCAGGTCCGTGTCCTGGA      120
                    ************************************************************

NM_001082437.2      CCCATTGCTCGCACGGGCCCCAGCAGGCGGCGCCAAGAGCTCTGCTCTGCCAGTGTGGGC      180
HH                  CCCATTGCTCGCACGGGCCCCAGCAGGCGGCGCCAAGAGCTCTGCTCTGCCAGTGTGGGC      180
HM                  CCCATTGCTCGCACGGGCCCCAGCAGGCGGCGCCAAGAGCTCTGCTCTGCCAGTGTGGGC      180
MH                  CCCATTGCTCTCACGGGCCCCAGCAGGCGGCGCCAAGAGCTCTGCTCTGCCAGTGTGGGC      180
MM                  CCCATTGCTCGCACGGGCCCCAGCAGGCGGCGCCAAGAGCTCTGCTCTGCCAGTGTGGGC      180
                    ********** *************************************************

NM_001082437.2      GGTGGCATCGGTCTCCGCAGTTGGCCTGAGCGGCGCGGGAGGCTGGTACGAGGCCTTGGC      240
HH                  GGTGGCATCGGTCTCCGCAGTTGGCCTGAGCGGCGCGGGAGGCTGGTACGAGGCCTTGGC      240
HM                  GGTGGCATCGGTCTCCGCAGTTGGCCTGAGCGGCGCGGGAGGCTGGTACGAGGCCTTGGC      240
MH                  GGTGGCATCGGTCTCCGCAGTTGGCCTGAGCGGCGCGGGAGGCTGGTACGAGGCCTTGGC      240
MM                  GGTGGCATCGGTCTCCGCAGTTGGCCTGAGCGGCGCGGGAGGCTGGTACGAGGCCTTGGC      240
                    ************************************************************

NM_001082437.2      TACCTCGGCGCCGGTGCAGGGCGCAGAGGACGTGCTGCTCTTCGCCCACACCGCCTCGGG      300
HH                  TACCTCGGCGCCGGTGCAGGGCGCAGAGGACGTGCTGCTCTTCGCCCACACCGCCTCGGG      300
HM                  TACCTCGGCGCCGGTGCAGGGCGCAGAGGACATGCTGCTCTTCGCCCACACCGCCTCGGG      300
MH                  TACCTCGGCGCCGGTGCAGGGCGCAGAGGACGTGCTGCTCTTCGCCCACACCGCCTCGGG      300
MM                  TACCTCGGCGCCGGTGCAGGGCGCAGAGGACGTGCTGCTCTTCGCCCACACCGCCTCGGG      300
                    ******************************* ****************************

NM_001082437.2      CCTGCCCTGGTGGGGCAGCATTCTCCTCACCACCGTGGTCCTGCGCGGGGCCGTCACACT      360
HH                  CCTGCCCTGGTGGGGCAGCATTCTCCTCACCACCGTGGTCCTGCGCGGGGCCGTCACACT      360
HM                  CCTGCCCTGGTGGGGCAGCATTCTCCTCACCACCGTGGCCCTGCGCGGGGCCGTCACACT      360
MH                  CCTGCCCTGGTGGGGCAGCATTCTCCTCACCACCGTGGTCCTGCGCGGGGCCGTCACACT      360
MM                  CCTGCCCTGGTGGGGCAGCATTCTCCTCACCACCGTGGTCCTGCGCGGGGCCGTCACACT      360
                    ************************************** *********************

NM_001082437.2      ACCCCTGGCTGCCTACCAGCACTACATCCTGGCCAAGGTGGAAAATTTGCAGCCAGAAAT      420
HH                  ACCCCTGGCTGCCTACCAGCACTACATCCTGGCCAAGGTGGAAAATTTGCAGCCAGAAAT      420
HM                  ACCCCTGGCTGCCTACCAGCACTACATCCTGGCCAAGGTGGAAAATTTGCAGCCAGAAAT      420
MH                  ACCCCTGGCTGCCTACCAGCACTACATCCTGGCCAAGGTGGAAAATTTGCAGCCAGAAAT      420
MM                  ACCCCTGGCTGCCTACCAGCACTACATCCTGGCCAAGGTGGAAAATTTGCAGCCAGAAAT      420
                    ************************************************************

NM_001082437.2      AAAAAACATTGCACGACATCTTAACCAAGAAGTTGCAGTTCGTGCACATCAGTTGGGATG      480
HH                  AAAAAACATTGCACGACATCTTAACCAAGAAGTTGCAGTTCGTGCACATCAGTTGGGATG      480
HM                  AAAAAACATTGCACGACATCTTAACCAAGAAGTTGCAGTTCGTGCACATCAGTTGGGATG      480
MH                  AAAAAACATTGCACGACATCTTAACCAAGAAGTTGCAGTTCGTGCCCATCAGTTGGGATG      480
MM                  AAAAAACATTGCACGACATCTTAACCAAGAAGTTGCAGTTCGTGCACATCAGTTGGGATG      480
                    ********************************************* **************

NM_001082437.2      GTCCAAGAGAGTTGCCAGGCTCACTTACCTA      511
HH                  GTCCAAGAGAGTTGCCAGGCTCACTTACCTA      511
HM                  GTCCAAGAGAGTTGCCAGGCTCACTTACCTA      511
MH                  GTCCAAGAGAGTTGCCAGGCTCACTTACCTA      511
MM                  GTCCAAGAGAGTTGCCAGGCTCACTTACCTA      511
                    *******************************
```

Figure S10. *COX18* gene (511-bp) demonstrative DNA sequence alignment between healthy and mastitis affected Holstein and Montbéliarde dairy cows together with the reference sequence found in GenBank gb|NM_001082437.2|. HH= Healthy Holstein; MH= Mastitic Holstein; HM= Healthy Montbéliarde and MM= Mastitic Montbéliarde.

```
NM_001192253.3      GTAACAACTTCTCCAGTATTCCCTCTCTGCCCCGAGGAAACCCGAGCCGGGCGCCGCGGG     60
HH                  GTAACAACTTCTCCAGTATTCCCTCTCTGCCCCGAGGAAACCCGAGCCGGGCGCCGCGGG     60
HM                  GTAACAACTTCTCCAGTATTCCCTCTCTGCCCCGAGGAAACCCGAGCCGGGCGCCGCGGG     60
MH                  GTAACAACTTCTCCAGTATTCCCTCTCTGCCCCGAGGAAACCCGAGCCGGGCGCCACGGG     60
MM                  GTAACAACTTCTCCAGTATTCCCTCTCTGCCCCGAGGAAACCCGAGCCGGGCGCCGCGGG     60
                    ******************************************************* ****

NM_001192253.3      CCCACCCCCAAAACCTCAAAGACTCCACTGGGGGCCCCTGCCCTGTCCCCTCTCACCGAT     120
HH                  CCCACCCCCAAAACCTCAAAGACTCCACTGGGGGCCCCTGCCCTGTCCCCTCTCACCGAT     120
HM                  CCCACCCCCAAAACCTCAAAGACTCCACTGGGGGCCCCTGCCCTGTCACCTCTCACCGAT     120
MH                  CCCACCCCCAAAACCTCAAAGACTCCACTGGGGGCCCCTGCCCTGTCCCCTCTCACCGAT     120
MM                  CCCACCCCCAAAACCTCAAAGACTCCACTGGGGGCCCCTGCCCTGTCCCCTCTCACCGAT     120
                    *********************************************** ************

NM_001192253.3      GCCACCACAAGCAGAAGCACTGCCCGCCGGTGCTGCCCGGCGGGGGGCTCCCGGCCACGC     180
HH                  GCCACCACAAGCAGAAGCACTGCCCGCCGGTGCTGCCCGGCGGGGGGCTCCCGGCCACGC     180
HM                  GCCACCACAAGCAGAAGCACTGCCCGCCGGTGCTGCCCGGCGGGGGGCTCCCGGCCACGC     180
MH                  GCCACCACAAGCAGAAGCACTGCCCGCCGGTGCTGCCCGGCGGGGGGCTCCCGGCCACGC     180
MM                  GCCACCACAAGCAGAAGCACTGCCCGCCGGTGCTGCCCGGCGGGGGGCTCCCGGCCACGC     180
                    ************************************************************

NM_001192253.3      CGCTGCTCTTCCACCCCCACACCAAGGGCTCCCAGATCCTCATGGACCTCAGCCACAAGG     240
HH                  CGCTGCTCTTCCACCCCCACACCAAGGGCTCCCAGATCCTCATGGACCTCAGCCACAAGG     240
HM                  CGCTGCTCTTCCACCCCCACACCAAGGGCTCCCAGATCCTCATGGACCTCAGCCACAAGG     240
MH                  CGCTGCTCTTCCACCCCCACACCAAGGGCTCCCAGATCCTCATGGACCTCAGCCACAAGG     240
MM                  CGCTGCTCTTCCACCCCCACACCAAGGGCTCCCAGATCCTCATGGACCTCAGCCACAAGG     240
                    ************************************************************

NM_001192253.3      CCGTCAAGAGGCAGGCCAGCTTCTGCAACGCCATCACCTTCAGTAACCGCCCGGTCCTCA     300
HH                  CCGTCAAGAGGCAGGCCAGCTTCTGCAATGCCATCACCTTCAGTAACCGCCCGGTCCTCA     300
HM                  CCGTCAAGAGGCAGGCCAGCTTCTGCAACGCCATCACCTTCAGTAACCGCCCGGTCCTCA     300
MH                  CCGTCAAGAGGCAGGCCAGCTTCTGCAACGCCATCACCTTCAGTAACCGCCCGGTCCTCA     300
MM                  CCGTCAAGAGGCAGGCCAGCTTCTGCAACGCCATCACCTTCAGTAACCGCCCGGTCCTCA     300
                    **************************** *******************************

NM_001192253.3      TCTATGAGCAAGTCAGGCTGAAGATCACCAAGAAGCAGTGCTGCTGGAGTGGGGCGCTGC     360
HH                  TCTATGAGCAAGTCAGGCTGAAGATCACCAAGAAGCAGTGCTGCTGGAGTGGGGCGCTGC     360
HM                  TCTATGAGCAAGTCAGGCTGAAGATCACCAAGAAGCAGTGCTGCTGGAGTGGGGCGCTGC     360
MH                  TCTATGAGCAAGTCAGGCTGAAGATCACCAAGAAGCAGTGCTGCTGGAGTGGGGCGCTGC     360
MM                  TCTATGAGCAAGTCAGGCTGAAGATCACCAAGAAGCAGTGCTGCTGGAGTGGGGCGCTGC     360
                    ************************************************************

NM_001192253.3      GGCTGGGCTTCACCAGCAAGGACCCGTCCCGCATCCACCCCGACTCGCTGCCCAAGTACG     420
HH                  GGCTGGGCTTCACCAGCAAGGACCCGTCCCGCATCCACCCCGACTCGCTGCCCAAGTACG     420
HM                  GGCTGGGCTTCACCAGCAAGGACCCGTCCCGCATCCACCCCGACTCGCTGCCCAAGTACG     420
MH                  GGCTGGGCTTCACCAGCAAGGACCCGTCCCGCATCCACCCCGACTCGCTGCCCAAGTACG     420
MM                  GGCTGGGCTTCACCAGCAAGGACCCGTCCCGCATCCACCCCGACTCGCTGCCCAAGTACG     420
                    ************************************************************

NM_001192253.3      CCTGCCCCGACCTGGTGTCCCAGAGCGGCTTCTGGGCCAAGGCGCTGCCTGAGGAG         476
HH                  CCTGCCCCGACCTGGTGTCCCAGAGCGGCTTCTGGGCCAAGGCGCTGCCTGAGGAG         476
HM                  CCTGCCCCGACCTGGTGTCCCAGAGCGGCTTCTGGGCCAAGGCGCTGCCTGAGGAG         476
MH                  CCTGCCCCGACCTGGTGTCCCAGAGCGGCTTCTGGGCCAAGGCGCTGCCTGAGGAG         476
MM                  CCTGCCCCGACCTGGTGTCCCAGAGCGGCTTCTGGGCCAAGGCGCTGCCTGAGGAG         476
                    ********************************************************
```

Figure S11. *NEURL1* gene (476-bp) demonstrative DNA sequence alignment between healthy and mastitis affected Holstein and Montbéliarde dairy cows together with the reference sequence found in GenBank gb| NM_001192253.3|. HH= Healthy Holstein; MH= Mastitic Holstein; HM= Healthy Montbéliarde and MM= Mastitic Montbéliarde.

```
NM_001098161.1      GCAAGAGCAAGATGGCCACTACCAAGCGAGTGTTGTATGTGGGTGGACTCGCAGAGGAGG      60
HH                  GCAAGAGCAAGATGGCCACTACCAAGCGAGTGTTGTATGTGGGTGGACTCGCAGAGGAGG      60
HM                  GCAAGAGCAAGATGGCCACTACCAAGCGAGTGTTGTATGTGGGTGGACTCGCAGAGGAGG      60
MH                  GCAAGAGCAAGATGGCCACTACCAAGCGAGTGTTGTATGTGGGTGGACTCGCAGAGGAGG      60
MM                  GCAAGAGCAAGATGGCCACTACCAAGCGAGTGTTGTATGTGGGTGGACTCGCAGAGGAGG      60
                    ************************************************************

NM_001098161.1      TGGATGACAAAGTTCTCCATGCTGCTTTTATCCCTTTTGGAGACATCACGGATATCCAGA      120
HH                  TGGATGACAAAGTTCTCCATGCTGCTTTTATCCCTTTTGGAGACATCACGGATATCCAGA      120
HM                  TGGATGACAAAGTTCTCCATGCTGCTTTTATCCCTTTTGGAGACATCACGGATATCCAGA      120
MH                  TGGATGACAAAGTTCTCCACGCTGCTTTTATCCCTTTTGGAGACATCACGGATATCCAGA      120
MM                  TGGATGACAAAGTTCTCCATGCTGCTTTTATCCCTTTTGGAGACATCACGGATATCCAGA      120
                    ******************* ****************************************

NM_001098161.1      TTCCTCTGGATTATGAAACAGAAAAGCACCGAGGATTTGCTTTTGTTGAATTTGAGTTGG      180
HH                  TTCCTCTGGATTATGAAACAGAAAAGCACCGAGGATTTGCTTTTGTTGAATTTGAGTTGG      180
HM                  TTCCTCTGGATTATGAAACAGAAAAGCACCGAGGATTTGCTTTTGTTGAATTTGAGTTGG      180
MH                  TTCCTCTGGATTACGAAACAGAAAAGCACCGAGGATTTGCTTTTGTTGAATTTGAGTTGG      180
MM                  TTCCTCTGGATTATGAAACAGAAAAGCACCGAGGATTTGCTTTTGTTGAATTTGAGTTGG      180
                    ************* **********************************************

NM_001098161.1      CAGAGGATGCTGCAGCAGCTATTGACAACATGAATGAATCTGAGCTCTTTGGACGGACAA      240
HH                  CAGAGGATGCTGCAGCAGCTATTGACAACATGAATGAATCTGAGCTCTTTGGACGGACAA      240
HM                  CAGAGGATGCTGCAGCAGCTATTGACAACATGAATGAATCTGAGCTCTTTGGACGGACAA      240
MH                  CAGAGGATGCTGCAGCAGCTATTGACAACATGAATGAATCTGAGCTCTTTGGACGGACAA      240
MM                  CAGAGGATGCTGCAGCAGCTATTGACAACATGAATGAATCTGAGCTCTTTGGACGGACAA      240
                    ************************************************************

NM_001098161.1      TTCGTGTCAATTTGGCAAAACCCATGAGGATTAGGAAGGCTCTTCTAGACCAGTTTGGT      300
HH                  TTCGTGTCAATTTGGCAAAACCCATGAGGATTAGGAAGGCTCTTCTAGACCAGTTTGGT      300
HM                  TTCGTGTCAATTTGGCAAAACCCATGAGGATTAGGAAGGCTCTTCCAGACCAGTTTGGT      300
MH                  TTCGTGTCAATTTGGCAAAACCCATGAGGATTAGGAAGGCTCTTCTAGACCAGTTTGGT      300
MM                  TTCGTGTCAATTTGGCAAAACCCATGAGGATTAGGAAGGCTCTTCTAGACCAGTTTGGT      300
                    ********************************************* **************

NM_001098161.1      CTGATGATGACTGGTTGAAGAAGT    324
HH                  CTGATGATGACTGGTTGAAGAAGT    324
HM                  CTGATGATGACTGGTTGAAGAAGT    324
MH                  CTGATGATGACTGGTTGAAGAAGT    324
MM                  CTGATGATGACTGGTTGAAGAAGT    324
                    ************************
```

Figure S12. *PPIE* gene (324-bp) demonstrative DNA sequence alignment between healthy and mastitis affected Holstein and Montbéliarde dairy cows together with the reference sequence found in GenBank gb| NM_001098161.1|. HH= Healthy Holstein; MH= Mastitic Holstein; HM= Healthy Montbéliarde and MM= Mastitic Montbéliarde.

```
NM_001076259.2      TCCAGCAATGCATATCTCTGTGATTCTGTTTTGTGCGCTCTGGTCTGCAGTGTCGGCGGA      60
HH                  TCCAGCAATGCATATCTCTGTGATTCTGTTTTGTGCGCTCTGGTCTGCAGTGTCGGCGGA      60
HM                  TCCAGCAATGCATATCTCTGTGATTCTGTTTTGTGCGCTCTGGTCTGCAGTGTCGGCGGA      60
MH                  TCCAGCAATGCATATCTCTGTGATTCTGTTTTGTGCGCTCTGGTCTGCAGTGTCGGCGGA      60
MM                  TCCAGCAATGCATATCTCTGTGATTCTGTTTTGTGCGCTCTGGTCTGCAGTGTCGGCGGA      60
                    ************************************************************

NM_001076259.2      GAACTCAGATGATTATGAGCTCATGTATGTGAATTTGGACAACGAAATAGACAATGGACT      120
HH                  GAACTCAGATGATTATGAGCTCATGTATGTGAATTTGGACAACGAAATAGACAATGGACT      120
HM                  GAACTCAGATGATTATGAGCTCATGTATGTGAATTTGGACAACGAGATAGACAATGGACT      120
MH                  GAACTCAGATGATTATGAGCTCATGTATGTGAATTTGGACAACGAAATAGACAATGGACT      120
MM                  GAACTCAGATGATTATGAGCTCATGTATGTGAATTTGGACAACGAAATAGACAATGGACT      120
                    ********************************************* **************

NM_001076259.2      CCATCCCACTGAGGACCCCACGCCGTGCGACTGCAGTCGTGAGAACTCCGAGTGGGACAA      180
HH                  CCATCCCACTGAGGACCCCACGCCGTGCGACTGCAGTCGTGAGAACTCCGAGTGGGACAA      180
HM                  CCATCCCACTGAGGACCCCACGCCGTGCGACTGCAGTCGTGAGAACTCCGAGTGGGACAA      180
MH                  CCATCCCACTGAGGACCCCACGCCGTGCGACTGCAGTCGTGAGAACTCCGAGTGGGACAA      180
MM                  CCATCCCACTGAGGACCCCACGCCGTGCGACTGCAGTCGTGAGAACTCCGAGTGGGACAA      180
                    ************************************************************

NM_001076259.2      GCTCTTCACCATGCTGGAGAACTCGCAGATGCGGGAGGGCATGCTGCTGCAGGCCACCGA      240
HH                  GCTCTTCACCATGCTGGAGAACTCGCAGATGCGGGAGGGCATGCTGCTGCAGGCCACCGA      240
HM                  GCTCTTCACCATGCTGGAGAACTCGCAGATGCGGGAGGGCATGCTGCTGCAGGCCACCGA      240
MH                  GCTCTTCATCATGCTGGAGAACTCGCAGATGCGGGAGGGCATGCTGCTGCAGGCCACCGA      240
MM                  GCTCTTCACCATGCTGGAGAACTCGCAGATGCGGGAGGGCATGCTGCTGCAGGCCACCGA      240
                    ******** ***************************************************

NM_001076259.2      CGTCATGCTCCGGGGCGAGCTGCAGAAGCTGCAGGCCGAGCTGGGCCGGCTGGAGGGAAG      300
HH                  CGTCATGCTCCGGGGCGAGCTGCAGAAGCTGCAGGCCGAGCTGGGCCGGCTGGAGGGAAG      300
HM                  CGTCATGCTCCGGGGCGAGCTGCAGAAGCTGCAGGCCGAGCTGGGCCGGCTGGAGGGAAG      300
MH                  CGTCATGCTCCGGGGCGAGCTGCAGAAGCTGCAGGCCGAGCTGGGCCGGCTGGAGGGAAG      300
MM                  CGTCATGCTCCGGGGCGAGCTGCAGAAGCTGCAGGCCGAGCTGGGCCGGCTGGAGGGAAG      300
                    ************************************************************

NM_001076259.2      CCTGCAGAAGCTGTGCGGGCCGGAGGCCCCCTCCGAGACCAGGCTGGCCCGGGCGCTGGA      360
HH                  CCTGCAGAAGCTGTGCGGGCCGGAGGCCCCCTCCGAGACCAGGCTGGCCCGGGCGCTGGA      360
HM                  CCTGCAGAAGCTGTGCGGGCCGGAGGCCCCCTCCGAGACCAGGCTGGCCCGGGCGCTGGA      360
MH                  CCTGCAGAAGCTGTGCGGGCCGGAGGCCCCCTCCGAGACCAGGCTGGCCCGGGCGCTGGA      360
MM                  CCTGCAGAAGCTGTGCGGGCCGGAGGCCCCCTCCGAGACCAGGCTGGCCCGGGCGCTGGA      360
                    ************************************************************

NM_001076259.2      CGACCTGCTGCAGGCGAGCCGCGATGCTGGCCGCCGGCTGGCGCGCCTGGAAGATGCTGG      420
HH                  CGACCTGCTGCAGGCGAGCCGCGATGCTGGCCGCCGGCTGGCGCGCCTGGAAGATGCTGG      420
HM                  CGACCTGCTGCAGGCGAGCCGCGATGCTGGCCGCCGGCTGGCGCGCCTGGAAGATGCTGG      420
MH                  CGAGCTGCTGCAGGCGAGCCGCGATGCTGGCCGCCGGCTGGCGCGCCTGGAAGATGCTGG      420
MM                  CGACCTGCTGCAGGCGAGCCGCGATGCTGGCCGCCGGCTGGCGCGCCTGGAAGATGCTGG      420
                    *** ********************************************************

NM_001076259.2      GGCGCTGCGACCGCAGGAGGAGGCGGGGCGGGCCCTGGGCGCGGTGCTGGAGGAGCTGCG      480
HH                  GGCGCTGCGACCGCAGGAGGAGGCGGGGCGGGCCCTGGGCGCGGTGCTGGAGGAGCTGCG      480
HM                  GGCGCTGCGACCGCAGGAGGAGGCGGGGCGGGCCCTGGGCGCGGTGCTGGAGGAGCTGCG      480
MH                  GGCGCTGCGACCGCAGGAGGAGGCGGGGCGGGCCCTGGGCGCGGTGCTGGAGGAGCTGCG      480
MM                  GGCGCTGCGACCGCAGGAGGAGGCGGGGCGGGCCCTGGGCGCGGTGCTGGAGGAGCTGCG      480
                    ************************************************************

NM_001076259.2      GCGGACGCGGGCCGATCTCCGAGCTGTGCAGGGCTGGGCAGCCAGCCGCTGGCTGCCGGC      540
HH                  GCGGACGCGGGCCGATCTCCGAGCTGTGCAGGGCTGGGCAGCCAGCCGCTGGCTGCCGGC      540
HM                  GCGGACGAGGGCCGATCTCCGAGCTGTGCAGGGCTGGGCAGCCAGCCGCTGGCTGCCGGC      540
MH                  GCGGACGCGGGCCGATCTCCGAGCTGTGCAGGGCTGGGCAGCCAGCCGCTGGCTGCCGGC      540
MM                  GCGGACGCGGGCCGATCTCCGAGCTGTGCAGGGCTGGGCAGCCAGCCGCTGGCTGCCGGC      540
                    ******* ****************************************************

NM_001076259.2      AGGTTGTGAAACAGCCATTTTATTCCCCATGCGTTCCAAGAAGATTTTTGCAAGCGTGCA      600
HH                  AGGTTGTGAAACAGCCATTTTATTCCCCATGCGTTCCAAGAAGATTTTTGCAAGCGTGCA      600
HM                  AGGTTGTGAAACAGCCATTTTATTCCCCATGCGTTCCAAGAAGATTTTTGCAAGCGTGCA      600
MH                  AGGTTGTGAAACAGCCATTTTATTCCCCATGCGTTCCAAGAAGATTTTTGCAAGCGTGCA      600
MM                  AGGTTGTGAAACAGCCATTTTATTCCCCATGCGTTCCAAGAAGATTTTTGCAAGCGTGCA      600
                    ************************************************************

NM_001076259.2      TCCGGTGACACCAATGA 617
HH                  TCCGGTGACACCAATGA 617
HM                  TCCGGTGACACCAATGA 617
MH                  TCCGGTGACACCAATGA 617
MM                  TCCGGTGACACCAATGA 617
                    *****************
```

Figure S13. *PTX3* gene (617-bp) demonstrative DNA sequence alignment between healthy and mastitis affected Holstein and Montbéliarde dairy cows together with the reference sequence found in GenBank gb|NM_001076259.2|. HH= Healthy Holstein; MH= Mastitic Holstein; HM= Healthy Montbéliarde and MM= Mastitic Montbéliarde. The asterisks represent the similarity.
